# 凝胶渗透色谱净化-气相色谱-串联质谱法同时测定不同配方体系化妆品中7种二甲基环硅氧烷

**DOI:** 10.3724/SP.J.1123.2021.11024

**Published:** 2022-06-08

**Authors:** Gengpeng XIAO, Lu YUAN, Chunli LUO, Xiang LUO, Yousheng HUANG

**Affiliations:** 1.江西省分析测试研究所, 江西 南昌 330029; 1. Analysis and Testing Research Institute of Jiangxi Province, Nanchang 330029, China; 2.江西省检验检测认证总院检测认证技术发展研究院, 江西 南昌 330052; 2. Institute of Development and Research, Jiangxi General Institute of Testing and Certification, Nanchang 330052, China

**Keywords:** 凝胶渗透色谱, 气相色谱-串联质谱, 二甲基环硅氧烷, 化妆品, gel permeation chromatography (GPC), gas chromatography-tandem mass spectrometry (GC-MS/MS), dimethylcyclosiloxanes (DMCs), cosmetics

## Abstract

鉴于当前化妆品中二甲基环硅氧烷的添加乱象,以及关于二甲基环硅氧烷在化妆品中安全风险评价的研究也未有实质性进展,因此建立适合不同配方体系化妆品中二甲基环硅氧烷的测定方法具有一定的理论和现实意义。基于此,建立了凝胶渗透色谱净化结合气相色谱-串联质谱测定不同配方体系化妆品中7种二甲基环硅氧烷的方法。方法采用乙酸乙酯-环己烷(1∶1, v/v)提取,凝胶渗透色谱净化,通过DB-5ms色谱柱(30.0 m×0.25 mm×0.25 μm)分离和气相色谱-串联质谱选择反应监测(SRM)模式进行确证和检测,以正十六烷为内标物内标法定量。分别对内标物、提取溶剂和净化方式的选择进行了优化。在最终确立的条件下,7种二甲基环硅氧烷在0.05~1.0 mg/L范围内线性良好,相关系数为0.994~0.998;方法的检出限(LOD, *S/N*=3)和定量限(LOQ, *S/N*=10)分别为0.04~0.08 mg/kg和0.12~0.24 mg/kg;针对不同配方体系的化妆品基质,进行了低、中、高3个添加水平的加标回收试验,目标物的加标回收率为85.3%*~*108.8%,相对标准偏差(RSD)为3.1%~9.4%。该方法操作简便,灵敏度高,重复性好,能够满足不同配方体系化妆品中7种二甲基环硅氧烷的测定要求。采用所建立的方法对市面上的化妆品进行检测,八甲基环四硅氧烷(D4)和十甲基环五硅氧烷(D5)均有不同程度的检出。该方法的建立将为我国化妆品中二甲基环硅氧烷的质量监督检查提供技术依据,有利于保障化妆品的安全,同时也为后续化妆品中二甲基环硅氧烷的健康安全风险评价提供了技术支撑。

## Abstract (en)

At present, the addition of dimethylcyclosiloxanes (DMCs) in cosmetics is being debated and no substantial progress has been made in their safety risk assessment because of the lack of a suitable analytical method. Therefore, it is of theoretical and practical significance to establish a method suitable for the determination of DMCs in cosmetics with different formulation systems. Accordingly, a method based on gel permeation chromatography (GPC) purification combined with gas chromatography-tandem mass spectrometry (GC-MS/MS) was developed for the determination of seven DMCs in cosmetics. The cosmetic samples were extracted by ethyl acetate-cyclohexane (1∶1, v/v), purified by gel permeation chromatography, separated on a DB-5ms column (30.0 m×0.25 mm×0.25 μm), confirmed and detected by gas chromatography-tandem mass spectrometry in the selected reaction monitoring (SRM) mode, and quantified by the internal standard method with *n*-hexadecane as the internal standard. Experiments were carried out using *n*-tetradecane, *n*-hexadecane, and *n*-octadecane as the internal standards, and based on the retention time in GPC and GC, *n*-hexadecane was found to be the suitable choice for further analyses. The extraction efficiency for the target compounds was tested in different solvents such as methanol, *n*-hexane, acetonitrile, ethyl acetate, and ethyl acetate-cyclohexane (1∶1, v/v). Given the high recovery, ethyl acetate-cyclohexane (1∶1, v/v) was selected as the extraction solvent for analyses. Among the three purification methods (analysis without purification, solid-phase extraction (SPE), and GPC purification), GPC was selected as the best method because of the minimal matrix interference to the target compounds. Under the optimized conditions, the seven DMCs showed good linearities in the range of 0.05-1.0 mg/L. The correlation coefficients (*r*) were 0.994-0.998, which were greater than the required of the specification (*r*≥0.990). The limits of detection (LODs, *S/N*=3) were 0.04-0.08 mg/kg, and the limits of quantification (LOQs, *S/N*=3) were 0.12-0.24 mg/kg. According to the cosmetic matrix in different formulation systems, standard addition recovery tests at three levels of low, medium, and high were carried out. The average recovery rates of the targets were 85.3%-108.8%. The relative standard deviations (RSDs, *n*=6) were 3.1%-9.4%. The established method was also employed for the analysis of cosmetics in the market, and octamethylcyclotetrasiloxane (D4) and decamethylcyclopentasiloxane (D5) were detected at various levels in the cosmetics. The method established in this study has the advantages of operational simplicity, high sensitivity, and good reproducibility, and it allows for the determination of seven DMCs in cosmetics with different formulation systems. The establishment of this method provides a basis for the quality supervision and inspection of DMCs in cosmetics in China, in addition to providing technical support for follow-up health and safety evaluation.

二甲基环硅氧烷(dimethylcyclosiloxanes, DMCs)是一类以硅氧烷为主链的环状化合物,主要包括六甲基环三硅氧烷(D3)、八甲基环四硅氧烷(D4)、十甲基环五硅氧烷(D5)、十二甲基环六硅氧烷(D6)、十四甲基环七硅氧烷(D7)、十六甲基环八硅氧烷(D8)、十八甲基环九硅氧烷(D9)等,是合成硅油、硅树脂、硅橡胶的主要原料。硅油和硅树脂因其良好的润滑性广泛添加于各类化妆品中,其合成过程中未反应完全的二甲基环硅氧烷单体也会一并进入化妆品产品中。同时相比硅油、硅树脂等大分子化合物,小分子的D4和D5等具有一定的挥发性,添加后可以使产品更易涂抹,用后皮肤能产生丝滑、清爽的感觉,在化妆品中逐渐有替代合成油脂和矿物油的趋势。

然而,近年来的研究显示,二甲基环硅氧烷类化合物在环境中具有一定的持久性和高生物富集性,对动物及人体内分泌系统、免疫系统、呼吸系统及神经系统均有不利影响,且具有特定的生殖毒性,容易导致生育能力受到损害,还有可能引起慢性中毒甚至增加致癌几率^[1⇓⇓⇓-5]^。鉴于此,国际社会对化妆品中二甲基环硅氧烷的关注日益密切。加拿大环境保护法案(CEPA)于2011年宣布禁止添加D4和D5到护肤品中;欧盟化学管理项目(REACH)将D4和D5列入限制物质,规定在淋洗类化妆品中D4和D5的添加量不允许超过0.1%;日本早在2010年就将D5加入了化学品审查清单,并对其安全性进行了持续评估^[6,7]^。相比欧美,我国现行的化妆品管理规章制度对于化妆品中二甲基环硅氧烷的添加管理仍处于滞后状态,随着消费者对化妆品安全的高度重视,对化妆品中二甲基环硅氧烷的准确测定就显得尤为重要。

目前,关于二甲基环硅氧烷含量测定的报道主要集中在有机硅材料和纺织助剂领域^[8⇓-10]^,主要采用气相色谱-氢火焰离子化法(GC-FID)、气相色谱-质谱法(GC-MS)、气相色谱-串联质谱法(GC-MS/MS)进行检测。其中,GC-FID因仅以保留时间为定性条件,在复杂基体中容易出现假阳性现象,且二甲基环硅氧烷在FID检测器的响应低,使得该方法灵敏度偏低。此外,二甲基环硅氧烷在FID检测器内燃烧时产生的二氧化硅也容易积聚在检测器喷嘴和集电极上,影响仪器灵敏度,需要频繁拆卸和清洗^[9]^。GC-MS虽然方法灵敏度和特异性较高,但难以阐明化合物的结构裂解信息,其定性准确度依旧较欠缺,同样也容易产生假阳性结果。与这两种方法相比,GC-MS/MS具有灵敏度高、特异性强等特点,目标物经过碰撞解离得到碎片离子,通过选择反应监测(SRM)可对化合物的结构加以确证,其定性、定量准确性高,已广泛应用于基质复杂、背景干扰严重的痕量化合物分析^[11]^。

在化妆品领域,目前国内外鲜有关于二甲基环硅氧烷检测的研究报道。化妆品包括水系、水包油(O/W)、油包水(W/O)、全油脂系、粉系等不同类型的配方体系,其制造原料和生产工艺截然不同,其基质组成、目标化合物的干扰也不尽相同,前处理方法也完全不一样。目前应用于化妆品领域的前处理方法主要有基质固相分散法(MSPD)、直接提取法、固相微萃取法(SPME)、固相萃取法(SPE)和凝胶渗透色谱法(GPC)等^[12⇓-14]^。其中,MSPD对分散剂的选择至关重要,同时对于复杂样品的净化效果还有待提升。SPME对纤维涂层的要求特别高,且使用寿命有限,需要定期更换,其重复性和萃取效率都有待提高。SPE和GPC作为样品净化的手段,已被证明是最为方便、有效的样品前处理净化技术,在国内外已经得到较为广泛的应用,已成为美国食品药物管理局(FDA)、美国分析化学协会(AOAC)、欧盟(EN)等机构的法定前处理方法^[15⇓-17]^。

鉴于此,本文在参考相关文献^[18⇓⇓-21]^的基础上,针对4类不同配方体系的化妆品,以乙酸乙酯-环己烷(1∶1, v/v)为提取溶剂,比较了直接提取法、SPE和GPC 3种不同前处理方法的提取效率和净化效果。最终以GPC前处理结合GC-MS/MS检测技术,建立了不同配方体系化妆品中7种二甲基环硅氧烷的测定方法,并将该方法成功应用于实际样品分析。

## 1 实验部分

### 1.1 仪器与试剂

TSQ-8000EVO气相色谱-串联质谱联用仪(美国Thermo Fisher Scientific公司); Free Style全自动凝胶色谱净化系统(德国LCTech公司); VORTEX 3型涡旋混合器(德国IKA公司); HC-3514高速离心机(科大创新公司); N-1001旋转蒸发仪(日本EYELA公司); N-EVAP 112氮吹仪(美国Organomation公司); KQ-500DE超声波清洗仪(昆山市超声仪器有限公司); AL204电子分析天平(瑞士Mettler Toledo公司)。

D3(纯度≥98%)、D4(纯度≥98%)、D5(纯度≥99%)、D6(纯度≥97%)、D7(纯度≥95%)、D8(纯度≥96%)、D9(纯度≥98%)标准品、正构十六烷烃(*n*-C_16_H_34_,纯度≥98%)均购于上海阿拉丁生化科技股份有限公司。环己烷、乙酸乙酯(色谱纯)购于北京迪科马科技有限公司;氯化钠、无水硫酸钠(分析纯)购于国药集团化学试剂有限公司。

### 1.2 标准溶液的配制

称取0.01 g(精确到0.0001 g)二甲基环硅氧烷对照品,置于100 mL容量瓶中,用乙酸乙酯-环己烷(1∶1, v/v)溶解并稀释至刻度、摇匀,即得100 mg/L标准储备液。

称取0.01 g(精确到0.0001 g)正构十六烷烃内标物,置于250 mL容量瓶中,用乙酸乙酯-环己烷(1∶1, v/v)溶解并稀释至刻度、摇匀,即得40 mg/L内标储备液。

选取各自配方体系的空白样品用乙酸乙酯-环己烷(1∶1, v/v)提取,经凝胶渗透色谱净化后,浓缩至2 mL即得空白基质提取液。

移取适量标准储备液用乙酸乙酯-环己烷(1∶1, v/v)和空白基质提取液分别稀释制备成质量浓度分别为0.05、0.1、0.2、0.5、1.0 mg/L和含内标物为0.2 mg/L的乙酸乙酯-环己烷溶剂标准工作溶液和基质匹配标准工作溶液。

### 1.3 仪器条件

气相色谱条件:色谱柱为DB-5ms型石英毛细管柱(30.0 m×0.25 mm×0.25 μm);以氦气(纯度>99.999%)为载气,柱流量为1.0 mL/min;分流进样,分流比为1∶5,进样量为1 μL;进样口温度为250 ℃,柱温采用程序升温,于50 ℃保持4 min,以15 ℃/min速率升温至240 ℃并保持5 min。

质谱条件:电子轰击电离(EI)源,电子轰击能量70 eV,传输线温度240 ℃,离子源温度260 ℃,采用SRM定量。7种二甲基环硅氧烷及内标物正十六烷的保留时间、定量离子和定性离子列于表1中。

**表1:** 二甲基环硅氧烷及内标物的保留时间、定量离子、定性离子、线性方程和相关系数(*r*)

Compound	CAS No.	*t*_R_/ min	Ion pairs (*m/z*)	CEs/eV	Linear range/ (mg/L)	Linear equation	*r*
Hexamethylcyclotrisiloxane (D3)	541-05-9	3.68	191.0/119.0^*^	20, 15	0.05-1.0	*y*=0.141*x*-0.771	0.994
			207.1/191.1				
Octamethylcyclotetrasiloxane (D4)	556-67-2	5.74	265.1/249.1^*^	10, 10	0.05-1.0	*y*=0.401*x*+0.432	0.995
			281.1/265.1				
Decamethylcyclopentasiloxane (D5)	541-02-6	7.98	267.0/251.1^*^	15, 10	0.05-1.0	*y*=0.320*x*-0.522	0.996
			355.1/267.1				
Dodecamethylcyclohexasiloxane (D6)	540-97-6	9.52	341.1/73.1^*^	15, 10	0.05-1.0	*y*=0.101*x*+0.418	0.996
			429.1/341.1				
Tetradecamethylcycloheptasiloxane (D7)	107-50-6	11.23	281.1/73.1^*^	10, 15	0.05-1.0	*y*=0.104*x*-0.106	0.994
			281.1/265.1				
Hexadecamethylcyclooctasiloxane (D8)	556-68-3	12.58	355.1/73.1^*^	10, 5	0.05-1.0	*y*=0.449*x*-0.785	0.997
			401.0/327.1				
Octadecamethylcyclononasiloxane (D9)	556-71-8	14.99	355.1/267.1^*^	10, 10	0.05-1.0	*y*=0.122*x*-0.333	0.998
			429.1/341.1				
*n*-Hexadecane (*n*-C_16_H_34_, IS)	554-76-3	13.41	57.1/29.1^*^	10, 5	-	-	-
			71.1/43.1				

* quantitation ion; *y*: peak area ratio of the quantitative ion of the analyte to the internal standard; *x*: mass concentration ratio of the analyte to the internal standard; -: no data.

### 1.4 样品的提取

#### 1.4.1 水溶性、O/W、W/O、全油脂系型化妆品

称取1.0 g(精确到0.001 g)样品于玻璃离心管中,加入20 μL内标储备液,涡旋混匀。

水溶性及O/W型化妆品:加入1 g氯化钠并于涡旋混合器涡旋2 min,再准确加入10 mL乙酸乙酯-环己烷(1∶1, v/v)涡旋混合。

W/O型化妆品:准确加入10 mL乙酸乙酯-环己烷(1∶1, v/v)涡旋分散后,加入1 g氯化钠涡旋。

全油脂系型化妆品:准确加入10 mL乙酸乙酯-环己烷(1∶1, v/v)涡旋分散。

室温下超声提取20 min,以4000 r/min离心5 min。上清液经无水硫酸钠脱水后过滤,滤液待凝胶色谱净化。

#### 1.4.2 粉系型化妆品(胭脂和眼影)

称取2.5 g(精确到0.0001 g)样品于玻璃离心管中,加入50 μL内标储备液,涡旋混匀。加入12 mL乙酸乙酯-环己烷(1∶1, v/v)涡旋分散后,于室温下超声提取20 min,以4000 r/min离心5 min。收集上清液于25 mL比色管中,向残渣中再加入12 mL乙酸乙酯-环己烷(1∶1, v/v)重复提取一次,合并上清液,定容至刻度。上清液待凝胶色谱净化。

### 1.5 样品的净化

未净化:移取5 mL 1.4节中的提取液于40 ℃氮吹浓缩至2.0 mL,过0.22 μm滤膜后供测定。

SPE净化:移取5 mL按1.4节中的提取液于40 ℃氮吹浓缩至2.0 mL,然后将其转移至预先用3 mL水、3 mL甲醇活化的Bond Elut C18-SPE柱(100 mg, 3 mL)上,接着用3 mL甲醇洗涤、真空抽干,再用10 mL乙酸乙酯-环己烷(1∶1, v/v)洗脱,收集洗脱液,最后于40 ℃氮吹浓缩至2.0 mL,过0.22 μm滤膜后供测定。

GPC净化:将1.4节中的提取液5 mL,通过满环进样方式转移至自动凝胶净化系统中,以聚苯乙烯凝胶填料(200~400目,Bio-Beads^TM^ S-X3)的填充柱为净化柱,以乙酸乙酯-环己烷(1∶1, v/v)为淋洗液,以5.0 mL/min流速进行洗脱,收集12~20 min洗脱液,于旋转蒸发仪上40 ℃浓缩至2.0 mL,过0.22 μm滤膜后以供测定。

## 2 结果与讨论

### 2.1 GC-MS/MS的优化

#### 2.1.1 色谱柱的选择

本文比较了3种不同极性的色谱柱VF-WAXms(30.0 m×0.25 mm×0.25 μm)、VF-1701ms(30.0 m×0.25 mm×0.25 μm)、DB-5ms(30.0 m×0.25 mm×0.25 μm)(极性顺序为VF-WAXms>VF-1701ms>DB-5ms)对化合物的分离情况。二甲基环硅氧烷为弱极性物质,其在VF-WAXms和VF-1701ms色谱柱上保留较弱。D3在VF-WAXms柱上的保留时间介于溶剂乙酸乙酯和环己烷之间,故容易受溶剂影响而无法定量;D3在VF-1701ms柱上的保留时间位于溶剂峰之后,但受到了峰拖尾的影响使定量不准确。采用DB-5ms柱时,D3保留较强,其保留时间为3.68 min,色谱图见图1,远离溶剂出峰影响,且对另外6种二甲基环硅氧烷的分离效果同样较好,同时在空白试剂的SRM色谱图中未见因固定液流失而产生的本底信号。因此本方法选择DB-5ms为分离色谱柱。

**图1:**
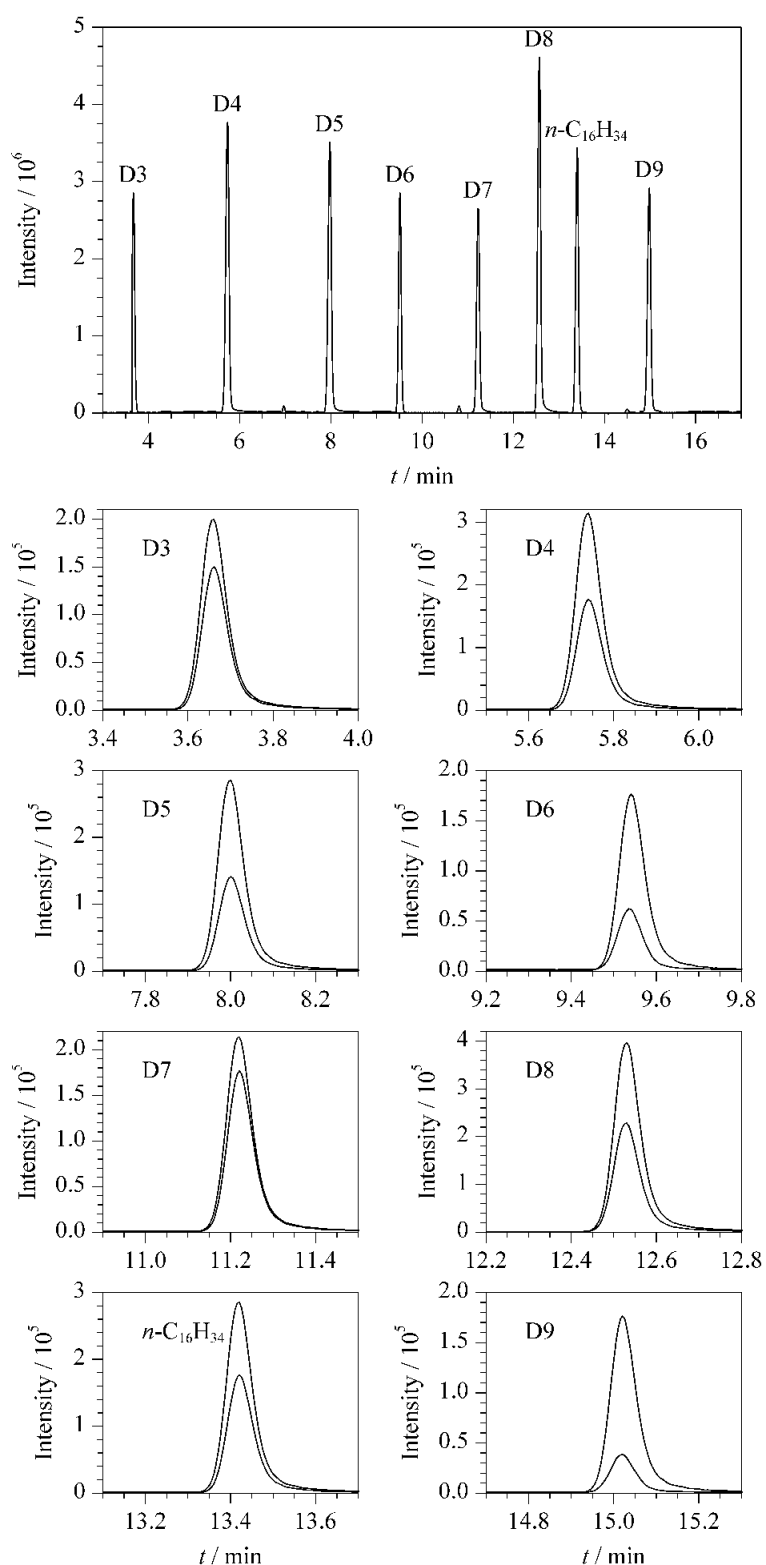
7种二甲基环硅氧烷及其内标的总离子流色谱图和SRM色谱图

#### 2.1.2 质谱参数的优化

在EI源(70 eV)模式下,将7种二甲基环硅氧烷和内标物的单标准溶液在全扫描模式下进行一级质谱扫描,每个化合物分别选取两组碎片离子*m/z*较大、强度较高的离子作母离子。母离子在氩气的碰撞能量传递下产生碎片离子,进行二级质谱扫描得到子离子,同样选取强度较高的离子作子离子。通过上述过程得到两组母离子和子离子的离子对,以其中强度大的离子对作为定量离子,另外一组离子对作为定性离子,并以此优化碰撞能量。从而得到各目标化合物的定量离子对、定性离子对和碰撞能量,详细结果见表1。

### 2.2 实验条件的优化

#### 2.2.1 内标物的选择

参考GB/T 28112-2011,本文选择正十四烷、正十六烷、正十八烷作为内标物,测试了其在GPC净化过程中的流出时间和气相色谱上的保留时间,以选择适合的内标物。结果表明,以正十四烷为内标物,其在GPC中的收集时间为23.5 min,远远超过7种二甲基环硅氧烷的收集时间。正十八烷在GPC中的收集时间小于7种二甲基环硅氧烷的收集时间,但在色谱柱上的保留时间大于D9,因此选取正十四烷、正十八烷为内标物均不合适。正十六烷在GPC中的收集时间处于7种二甲基环硅氧烷的收集时间范围内,且在色谱柱上的保留时间也介于D8~D9之间,与目标物的提取和测定的时间范围基本保持一致,因此选取正十六烷为测定内标物。

#### 2.2.2 提取溶剂的选择

二甲基环硅氧烷易溶于乙酸乙酯、正己烷、甲醇、乙腈、乙酸乙酯-环己烷(1∶1, v/v)等有机溶剂。为选择最适合的提取溶剂,本文选取基质最为复杂的W/O型化妆品为研究对象进行空白样品添加回收试验。分别用以上溶剂进行提取,计算回收率。7种二甲基环硅氧烷在不同溶剂中的回收率结果见图2,以乙酸乙酯-环己烷(1∶1, v/v)提取时的回收率为85.6%~96.7%。乙酸乙酯、甲醇、乙腈对D3及D4的提取效果较好,但对大环二甲基环硅氧烷特别是D9的提取效果较差,其回收率为68.0%~75.0%;而正己烷对大环二甲基环硅氧烷特别是D9的提取效果好,但对D3的提取效率稍差。这可能是因为随着环数的增加,D3~D9这7种化合物的极性逐渐减小,根据相似相溶原理,极性溶剂对相对极性较大的小环化合物提取效率较高,非极性溶剂对相对极性较小的大环化合物提取效果较好,而乙酸乙酯-环己烷(1∶1, v/v)由极性溶剂和非极性溶剂相互混合,能够对小环及大环化合物同时展现出较好的提取效果。综上所述,乙酸乙酯-环己烷(1∶1, v/v)是本文的最佳提取溶剂。

**图2:**
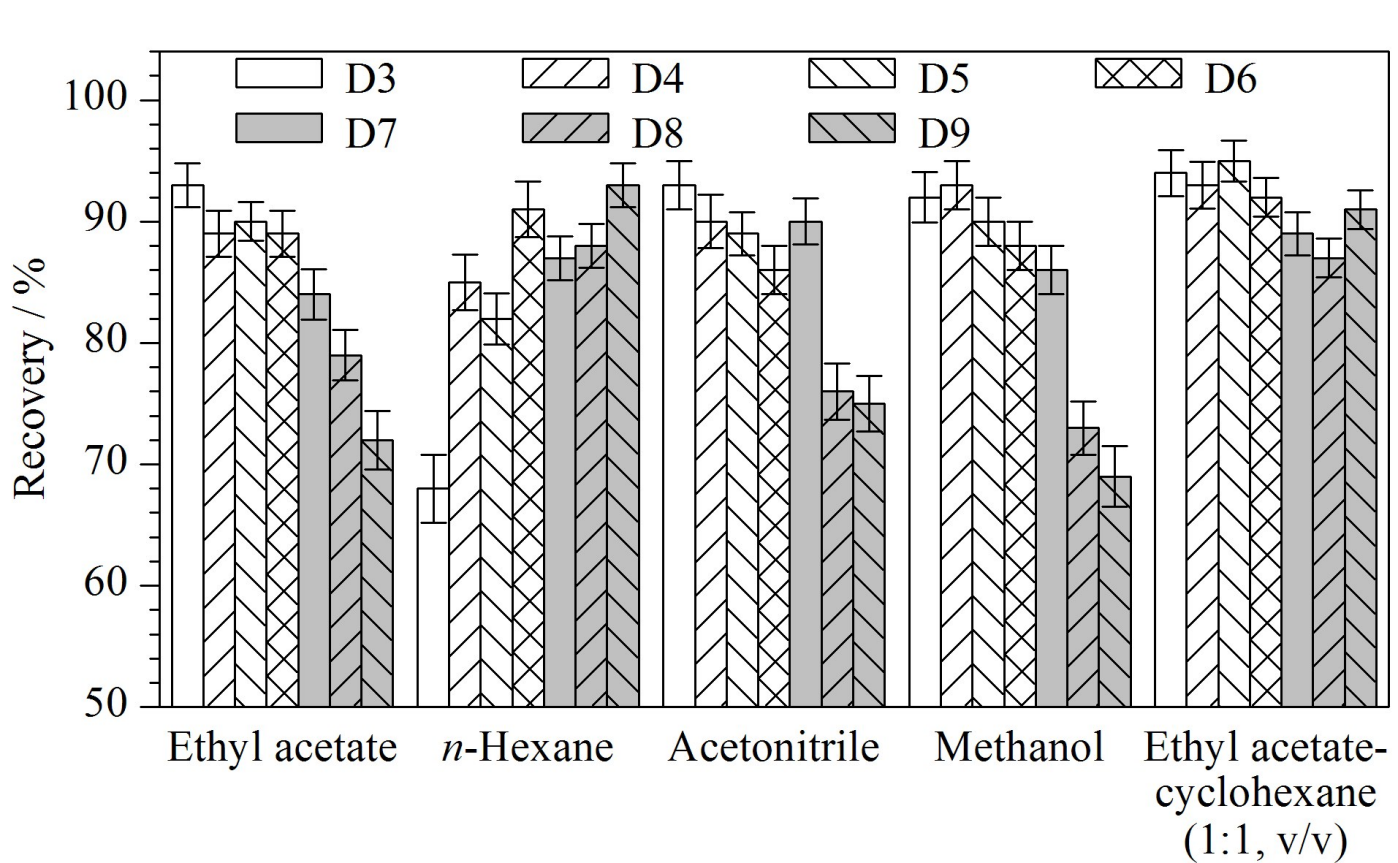
不同提取溶剂对7种DMCs回收率的影响(*n*=3)

#### 2.2.3 净化过程的优化

化妆品配方组成复杂,通常是由数十种不同的功能性原料通过特殊生产工艺复配而成。体系基质的复杂性,无疑对净化方式提出了严峻的挑战。基于此,本文对比了未净化、C18-SPE柱净化和GPC净化3种不同方式对样品的净化效果。

结果表明,未净化样品基质效应强、干扰大,造成回收率低。C18-SPE柱属于反相净化柱,可以很好地除去样品中的极性组分,但是化妆品中非极性油脂的影响使得柱子很容易过载,同时受高分子聚合物的影响容易造成堵柱,从而使得测定重复性差。另外对于中等极性的D3,其回收率也偏低(仅65.3%)。GPC净化是根据相对分子质量大小的不同,化合物在凝胶渗透色谱柱上的保留时间不同达到分离净化的目的,适用于色素、油脂、糖类及其高分子化合物的有效净化,而且GPC可实现仪器自动净化,重复性好,各化合物的回收率均在85%以上。在本实验中,二甲基环硅氧烷在紫外下几乎无吸收,无法通过GPC仪器配置的紫外检测器确定收集时间,为此我们通过多次的标准溶液洗脱和分段收集结合GC-MS/MS检测,最终确定收集时间段为12.0~20.0 min。

### 2.3 基质效应

基质效应是质谱分析中难以回避的问题,严重时直接影响质谱定量结果的准确性。为了评估基质对实验的影响,本文参考相关文献^[22]^提出的评估方法,分别以乙酸乙酯-环己烷(1∶1, v/v)溶液和空白基质样品溶液配制标准溶液,并绘制标准曲线,以二者线性回归方程的斜率比(*K*_基质_/*K*_溶剂_)来评定化妆品基质的基质效应。一般来说,*K*_基质_/*K*_溶剂_<0.85表现为基质抑制效应;*K*_基质_/*K*_溶剂_>1.15,表现为基质增强效应;0.85≤*K*_基质_/*K*_溶剂_≤1.15,基质效应可以忽略。结果表明,D3、D4在O/W和W/O体系中的*K*_基质_/*K*_溶剂_>1.15,表现为基质增强效应,可能的原因是O/W和W/O为成分更为复杂的乳化体系。其余化合物在全部体系的化妆品中*K*_基质_/*K*_溶剂_介于0.85~1.15之间,基质效应可忽略。故为提高分析的准确性,对于O/W和W/O体系的化妆品在测定D3和D4时应该采用空白基质标准曲线进行定量分析,而测定O/W和W/O体系化妆品中其他5种二甲基环硅氧烷和其他配方体系化妆品中7种二甲基环硅氧烷均可以采用溶剂标准曲线进行定量。

### 2.4 方法验证

本文参考中华人民共和国食品药品监督管理局食药监许[2010]455号文件《化妆品中禁用物质和限用物质检测方法验证技术规范》进行方法验证,验证的内容包括:线性及线性范围、检出浓度和定量浓度、检出限和定量限、精密度、回收率、稳定性。

#### 2.4.1 线性范围和检出限

在1.3节中所述的仪器条件下对各化合物相应的标准溶液进行测定,每份标准样品测定3次,取峰面积平均值,并以标准物质质量浓度和内标物质量浓度的比值为横坐标(*x*),标准物质定量离子峰面积和内标物定量离子峰面积的比值为纵坐标(*y*)进行线性回归,相关参数结果列于表1中。结果表明,7种二甲基环硅氧烷在其对应的范围内均呈现良好的线性关系,其相关系数为0.994~0.998,符合方法验证规范要求。以3倍和10倍空白样品噪声对应的浓度确定为方法的检出浓度和定量浓度,其结果分别为0.01~0.02 mg/L和0.03~0.06 mg/L,并由此通过计算得出7种二甲基环硅氧烷在不同配方体系中的LOD和LOQ,分别为0.04~0.08 mg/kg和0.12~0.24 mg/kg(见表2)。

**表2:** 不同配方体系化妆品中的二甲基环硅氧烷检出限和定量限

Type	Sample	*C*_Detected_/(mg/L)	*C*_Quantitative_/(mg/L)	LOD/(mg/kg)	LOQ/(mg/kg)	
O/W cosmetics	hand cream	0.01	0.03	0.04	0.12	
W/O cosmetics	foundation primer	0.02	0.06	0.08	0.24	
Full oil type cosmetics	lip stick	0.01	0.03	0.04	0.12	
Powdered cosmetics	rouge	0.01	0.03	0.04	0.12	

O/W: oil in water; W/O: water in oil.

#### 2.4.2 回收率和精密度

采用空白样品加标的方式对7种二甲基环硅氧烷的回收率进行试验,分别于空白样品中加入低、中、高3个水平,按照1.4节中所述的处理方法进行处理并进样测定6次,计算平均回收率和精密度,结果见表3和表4。结果表明,二甲基环硅氧烷在4类不同配方体系的化妆品中的平均回收率为85.3%~108.8%,符合方法验证规范要求的85%~115%。RSD为3.1%~9.4%,同样均符合方法验证规范要求的≤10%。

**表3:** 不同配方体系化妆品中7种二甲基环硅氧烷的加标回收率(*n*=6)

Compound	Hand cream				Foundation primer				Lip stick				Rouge							
	0.5 mg/kg	1.0 mg/kg	3.0 mg/kg		0.5 mg/kg	1.0 mg/kg	3.0 mg/kg		0.5 mg/kg	1.0 mg/kg	3.0 mg/kg		0.5 mg/kg	1.0 mg/kg		3.0 mg/kg				
D3	86.7	92.3	87.3		89.7	93.8	86.6		88.7	91.4	86.4		88.1	91.1		89.1	
D4	87.4	88.5	89.0		85.8	92.7	87.1		87.6	87.3	89.2		86.4	87.9		89.8	
D5	90.3	85.3	93.5		86.9	94.6	91.5		91.2	85.7	90.5		91.1	87.1		93.7	
D6	85.6	93.5	100.9		86.3	92.1	108.8		86.4	91.5	89.9		86.2	94.2		103.7	
D7	88.2	101.8	107.3		87.2	88.8	105.3		86.2	106.8	101.2		87.3	103.7		101.6	
D8	85.8	96.5	91.7		85.9	86.7	92.7		88.7	91.5	88.6		85.6	94.2		90.3	
D9	91.1	103.8	106.2		86.1	90.8	103.8		90.7	104.8	101.4		90.1	89.8		103.1	

**表4:** 不同配方体系化妆品中7种二甲基环硅氧烷的日内精密度和日间精密度(*n*=6)

Compound	Hand cream				Foundation primer				Lip stick				Rouge							
	0.5 mg/kg	1.0 mg/kg	3.0 mg/kg		0.5 mg/kg	1.0 mg/kg	3.0 mg/kg		0.5 mg/kg	1.0 mg/kg	3.0 mg/kg		0.5 mg/kg	1.0 mg/kg		3.0 mg/kg				
D3	4.5(5.2)^#^	4.1(4.7)	3.5(4.2)		6.5(5.8)	4.6(4.2)	5.5(7.2)		5.8(5.1)	4.0(5.3)	3.5(5.3)		4.1(5.6)	5.5(7.2)		3.5(4.6)	
D4	8.2(6.9)	3.8(5.3)	4.1(4.4)		6.1(5.8)	6.5(5.3)	6.5(5.7)		4.5(5.0)	3.5(5.1)	4.5(5.6)		6.5(7.2)	4.1(7.0)		4.7(5.1)	
D5	5.5(6.7)	6.5(5.1)	5.5(3.9)		5.5(4.7)	5.1(6.5)	4.8(5.2)		4.3(6.2)	3.1(4.7)	4.8(5.1)		7.0(6.2)	3.5(4.8)		4.2(5.5)	
D6	6.2(7.3)	4.5(4.2)	4.4(5.6)		7.5(9.2)	6.5(7.4)	6.3(7.2)		5.7(5.1)	4.5(5.9)	3.5(4.2)		3.8(5.0)	4.9(5.5)		6.5(5.0)	
D7	4.6(6.3)	5.5(6.5)	5.1(7.5)		6.5(9.4)	6.1(8.2)	7.5(6.6)		6.5(5.3)	3.7(5.1)	3.9(5.8)		5.5(5.1)	3.7(5.2)		3.5(5.4)	
D8	5.6(9.2)	4.1(5.6)	3.5(4.1)		5.7(6.6)	5.5(7.4)	6.5(5.4)		5.5(5.1)	4.1(6.2)	4.0(6.3)		6.5(7.8)	4.1(5.2)		3.5(6.1)	
D9	4.8(6.4)	4.8(7.2)	3.1(4.3)		6.5(5.4)	6.7(8.4)	5.7(6.8)		4.5(5.7)	5.5(7.0)	5.5(7.2)		5.5(5.9)	3.5(5.3)		4.8(6.2)	

# Intra-day precision (inter-day precision).

#### 2.4.3 稳定性

选取2.4.2节高、低两个添加水平的W/O型化妆品,经1.4节和1.5节处理后的溶液在4 ℃避光保存,每天测定一次、连续测试7 d,计算各目标化合物含量的RSD。结果表明,在两个添加水平样品的提取液中,各化合物含量的RSD均小于10%,说明本实验的提取液在7 d内保持稳定,有利于保证实验结果的准确可靠。

### 2.5 实际样品检测

在市场和电商平台上随机购买30个不同配方体系的化妆品,包括5份洗发水、5份精华液、5份O/W乳液、5份W/O防晒霜、5份口红、5份胭脂,分别按照1.4节的方法进行前处理,并根据待测组分含量的高低对样品溶液进行适当稀释,使其浓度落在标准曲线范围内,然后测定二甲基环硅氧烷的含量。其中,3份W/O防晒霜、2份O/W乳液、2份口红、1份洗发水和1份胭脂共9份化妆品检出二甲基环硅氧烷,检出率为30%,主要检出物质为D4和D5,含量为38.3~24366 mg/kg。与化妆品备案配方对比发现,其中4份化妆品为人为添加,其种类与化妆品备案配方表一致,另外5份化妆品在备案配方表中未显示添加,这可能来源于配方中所列有机硅原料合成过程中残留的单体。

## 3 结论

本文以不同配方体系的化妆品为研究对象,对不同的前处理方法进行了优化,建立了凝胶渗透色谱净化结合气相色谱-串联质谱测定化妆品中7种二甲基环硅氧烷含量的方法。该方法操作简便,灵敏度高,回收率高,重复性好,并成功用于实际样品的检测,可为化妆品中二甲基环硅氧烷的安全评价提供技术支撑,也有助于化妆品公司和监管部门对化妆品的产品质量进行监督检查。
